# Transforming Growth Factor β_1_ Induces the Expression of Collagen Type I by DNA Methylation in Cardiac Fibroblasts

**DOI:** 10.1371/journal.pone.0060335

**Published:** 2013-04-01

**Authors:** Xiaodong Pan, Zhongpu Chen, Rong Huang, Yuyu Yao, Genshan Ma

**Affiliations:** Department of Cardiology, Zhongda Hospital, Medical School of Southeast University, Nanjing, Jiangsu, China; University of Bergen, Norway

## Abstract

Transforming growth factor-beta (TGF-β), a key mediator of cardiac fibroblast activation, has a major influence on collagen type I production. However, the epigenetic mechanisms by which TGF-β induces collagen type I alpha 1 (COL1A1) expression are not fully understood. This study was designed to examine whether or not DNA methylation is involved in TGF-β-induced COL1A1 expression in cardiac fibroblasts. Cells isolated from neonatal Sprague-Dawley rats were cultured and stimulated with TGF-β_1_. The mRNA levels of COL1A1 and DNA methyltransferases (DNMTs) were determined via quantitative polymerase chain reaction and the protein levels of collagen type I were determined via Western blot as well as enzyme-linked immunosorbent assay. The quantitative methylation of the COL1A1 promoter region was analyzed using the MassARRAY platform of Sequenom. Results showed that TGF-β_1_ upregulated the mRNA expression of COL1A1 and induced the synthesis of cell-associated and secreted collagen type I in cardiac fibroblasts. DNMT1 and DNMT3a expressions were significantly downregulated and the global DNMT activity was inhibited when treated with 10 ng/mL of TGF-β_1_ for 48 h. TGF-β_1_ treatment resulted in a significant reduction of the DNA methylation percentage across multiple CpG sites in the rat COL1A1 promoter. Thus, TGF-β_1_ can induce collagen type I expression through the inhibition of DNMT1 and DNMT3a expressions as well as global DNMT activity, thereby resulting in DNA demethylation of the COL1A1 promoter. These findings suggested that the DNMT-mediated DNA methylation is an important mechanism in regulating the TGF-β_1_-induced COL1A1 gene expression.

## Introduction

Cardiac fibrosis is characterized by the induction of profibrotic growth factors and activation of cardiac fibroblasts (CFs), which have an important function in the development of myocardial remodeling process [1]–[3]. Activated CFs change their phenotype and differentiate into myofibroblasts as characterized by the expression of α-smooth muscle actin and production of extracellular matrix (ECM) proteins [1], [4]. Collagen type I and other ECM proteins can be excessively deposited, which occurs in fibrotic diseases, thereby resulting in organ dysfunction and failure. The regulation of collagen type I gene expression in healthy tissues during development and wound healing as well as dysregulation in fibrosis has been the subject of comprehensive studies. Among various soluble molecules that induce collagen type I expression, transforming growth factor-beta (TGF-β) is one of the most extensively studied [5], [6]. TGF-β and its downstream Smad signaling have an essential function in tissue fibrosis although various different fibrogenic factors have been documented. For instance, TGF-β_1_ is a key mediator of CF activation and has a major influence on ECM production [7]. Enhanced TGF-β_1_ expression is often accompanied by increased collagen synthesis, deposition, and myocardial fibrosis [8].

Collagen type I, the major component of ECM, forms a characteristic triple-helix structure composed of two a1 (I) chains and one a2 (I) chain, which are encoded by COL1A1 and COL1A2 genes, respectively, in which the coordinated transcription rates of these genes ensure a 2∶1 ratio [9]. The synthesis of different collagen type I polypeptides is controlled by two separate pathways: the TGF-β_1_ activation protein pathway and the Smad signaling pathway [10]. The complete transcription of both genes is required for collagen type I synthesis. In this paper, we focused on the COL1A1 gene. COL1A1 gene regulation is regulated by the TGF-β activator protein binding directly to the TGF-β *cis*-element and original works demonstrated that TGF-β-responsive sequences are located between −174 and −84 bp from the transcription start site [11]. Although the transcriptional regulation of the COL1A1 gene has been widely studied [12], [13], little information regarding the epigenetic regulation of this aspect of COL1A1 expression is available.

The main epigenetic mechanisms of gene regulation are DNA methylation and histone modification [14]. The DNA methylation pattern is an important component of the regulatory mechanisms of gene expression [15]–[17]. DNA methylation is a covalent modification, in which cytosine is methylated in a reaction that is catalyzed by DNA methyltransferases (DNMTs) and S-adenosyl methionine serves as a methyl donor [18]. Like many other genes, DNA methylation of regulatory and structural regions of Type I collagen gene causes its downregulation. Previous studies reported that surrounding the start site of murine COL1A1 promoter is methylated in undifferentiated embryonal cells and demethylated in collagen-producing and –nonproducing differentiated cells [19]. Methylation in the promoter region suppresses the COL1A1 gene expression in cultured 3T3 and F9 cells [20]. This COL1A1 gene expression suppression is associated with increased DNA methylation after normal human lung fibroblasts are transformed by SV40 [21]. Recent studies found that DNA hypermethylation in the promoter regions is of great importance for the age-associated decrease in the COL1A1 gene expression in the periodontal ligament [22]. COL1A1 gene promoter regions are frequently methylated in primary renal cell tumors [23]. The hypermethylation of CpG sites in the COL1A1 promoter may reduce collagen synthesis at the transcriptional level in myopic scleras [24]. However, the epigenetic regulation of COL1A1 in the heart has not been widely studied. The present study aimed to investigate the methylation regulation of COL1A1 in cultured CFs that were treated with TGF-β_1_. The methylation status of the COL1A1 promoter regions was evaluated and the DNMT expression was analyzed.

## Materials and Methods

### Ethics Statement

All procedures in the present study were conducted in accordance with the National Instituted of Health Guide for the Care and Use of Laboratory Animals and approved by the Care of Experimental Animals Committee of the Southeast University (Approval ID: SYXK-2011.3923).

### Reagents and Antibodies

Recombinant human TGF-β_1_ was purchased from Peprotech (London, UK). TGF-β-neutralizing antibody was from R&D Systems (Minneapolis, MN, USA) and 5-aza-2′-deoxycytidine (5-aza-dC) was obtained from Sigma Aldrich (St. Louis, MO, USA). Wizard® SV Genomic DNA Purification System was purchased from Promega (Madison, WI, USA). Trizol reagent was from Invitrogen (Carlsbad, CA, USA) and reverse transcription reagents were from Fermentas (Hanover, MD, USA). Nuclear extraction kit was from KeyGEN Biotech (Nanjing, China) and DNMT activity assay kit was purchased from GENMED Scientifics (Shanghai, China). ELISA detection kit of collagen type I was from BioLeaf (Shanghai, China). Mouse monoclonal antibodies against rat vimentin, desmin, and Factor VIII were purchased from Boster Biological Technology (Wuhan, China). Goat anti-COL1A1 polyclonal antibody, anti-GAPDH polyclonal antibody and horseradish peroxidase-conjugated secondary antibody were from Santa Cruz Biotechnology (Santa Cruz, CA, USA).

### Cell culture

CFs from neonatal (1 to 3 days old) Sprague-Dawley rats (Yangzhou Laboratory Animal Center, China) were isolated according to the standard protocol [25]. Briefly, 40 to 50 neonatal hearts were rapidly excised from anesthetized animals (Pentobarbital 5 mg/kg, IP), minced, and placed in a collagenase/trypsin digestion solution. After the hearts were subjected to five to six digestion periods, 10% fetal bovine serum was added to neutralize trypsin and the accumulated cell suspension was stored in an ice bath. The dissociated cells were collected via centrifugation at 300×*g* for 5 min, resuspended in Dulbecco's modified Eagle's medium (DMEM), and supplemented with 10% fetal bovine serum as well as 1% penicillin-streptomycin. The cell suspension was then kept for 60 min at 37°C in a humidified atmosphere that contains 5% CO_2_ to allow noncardiomyocytes (mostly CFs) to attach to the dishes. The remaining cardiomyocytes in the medium were discarded. The attached CFs were further cultured to confluence, and then passaged at 1∶3 dilution. Second-passage CFs were used throughout the experiment.

### Immunocytochemistry

The cells were seeded onto cover slips in six-well dishes and allowed to attach overnight in a medium that contains 10% serum. The cells were rendered quiescent in serum-free medium for another 12 h. The medium was removed and the cells were rinsed with PBS then fixed with 4% paraformaldehyde. The cells were permeabilized with 0.1% Triton X-100 and incubated overnight with primary antibodies against vimentin, desmin, and Factor VIII (1∶200) at 4°C. The cells were rinsed with phosphate-buffered saline (PBS), and then incubated with biotinylated secondary antibodies. The antibody binding was visualized using 3,3′-diaminobenzidine tetrahydrochloride before the cells were briefly counterstained with Mayer's hematoxylin. Visualization was performed under an inverted microscope.

### Quantitative real-time polymerase chain reaction (PCR)

The mRNA levels of COL1A1 and three DNMTs were determined via quantitative real-time PCR to assess the effect of TGF-β_1_ on COL1A1 expression in CFs. After the experimental treatment was performed, total RNA was isolated using Trizol reagent, and then reverse transcribed to single-strand cDNA using reverse transcription reagents according to the manufacturer's instructions. Quantitative real-time PCR experiments were performed using the IQ SYBR Green Supermix (Bio-Rad) and BIO-RAD MJ Mini Opticon Real-Time PCR System. The resulting amplification and melt curves were analyzed to ensure the identity of the specific PCR product. Threshold cycle values were used to calculate the fold change in the transcript levels by using the 2^-ΔΔCt^ method. The relative mRNA expression levels were normalized to the actin gene. The primer sequences are listed as follows: COL1A1, forward primer CAGTCGATTCACCTACAGCACG and reverse primer GGGATGGAGGGAGTTTACACG; DNMT1, forward primer ACCACGCCGACATCAACCT and reverse primer TCCTCCACAGCCAGAAAACAC; DNMT3a, forward primer GGCCCATTCGATCTGGTGA and reverse primer CTTGGCTATTCTGCCGTGTTC; DNMT3b, forward primer GGTGCGTCGTTCAGGCAGT and reverse primer TCCTCATCTTCCCCTCGGTC; actin, forward primer CCCATCTATGAGGGTTACGC and reverse primer TTTAATGTCACGCACGATTTC.

### Western blot analysis

Cell-associated collagen type I was determined via Western blot. After the experimental treatment was performed, the cells were lysed with lysis buffer and the cell extract protein concentration was quantified via the bicinchoninic acid assay. Equal amounts of protein (30 µg) of the lysates were separated on 10% polyacrylamide gels (Bio-Rad). The separated proteins were then transferred into polyvinylidene difluoride membranes, which were blocked for 1 h at room temperature by using 5% skimmed milk or 2% bovine serum albumin in TBST solution (10 mM Tris-HCl, 150 mM NaCl, and 0.05% Tween 20) and subsequently incubated overnight at 4°C with primary antibody (1∶1000). The membranes were then washed and incubated with horseradish peroxidase-conjugated secondary antibody (1∶5000) for 1 h at room temperature. Immunoreactive bands were visualized using an enhanced chemiluminescence reagent and quantified via scanning densitometry. The results were expressed relative to the band density of GAPDH, which was used as a loading control.

### Enzyme-linked immunosorbent assay (ELISA)

The cells were cultured until near confluence was reached, and then starved for 12 h in serum-free DMEM. After the experimental treatment was performed, the supernatants were collected from the cell cultures and frozen at–80°C before use. Collagen type I secretion in the culture supernatants of CFs was determined via ELISA by using commercially available kits according to the manufacturer's instructions. Absorbance was determined at 450 nm by using a microplate reader. The results were compared with a standard curve, which was constructed by titrating standards.

### Nuclear DNMT activity assay

CFs were starved for 12 h in serum-free DMEM, and then stimulated using 10 ng/mL of TGF-β_1_ for 48 h. Nuclear protein was extracted using a nuclear extraction kit. Approximately 20 µg of nuclear protein was used in the DNMT activity assay, which was performed using a DNMT activity assay kit according to the manufacturer's instructions.

### DNA methylation analysis

Genomic DNA was extracted from the cultured cells by using Wizard® SV Genomic DNA Purification System according to the manufacturer's instructions. DNA concentration and purity were determined based on the absorbance at 260 and 280 nm. A total of 1 µg of genomic DNA from each sample was bisulfite-treated using the EZ-96 DNA methylation kit (Zymo Research) according to the manufacturer's instructions. Sequenom MassARRAY platform (CapitalBio, Beijing, China), which was composed of matrix-assisted laser desorption/ionization time-of-flight (MALDI-TOF) mass spectrometry and combined with RNA base-specific cleavage was used to analyze COL1A1 methylation quantitatively (Gen-Bank Accession Number: NM_053304.1). PCR primers were designed using Methprimer (http://www.urogene.org/methprimer/). For each reverse primer, an additional T7 promoter tag for in vivo transcription was added, whereas a 10 m tag on the forward primer was used to adjust melting temperature differences. We used the following primers based on the reverse complementary strands of COL1A1-promoter 1 (5′-aggaagagagTTGTAAAGGTGTTTTGTTTGATTTTT-3′ and 3′-cagtaatacgactcactatagggagaaggctAACCTCTACAATCTCCCTCTACCAC-5′) and COL1A1-promoter 2 (5′-aggaagagagTTTGGAATTTATTGTTTTTTTGGTT-3′ and 3′-cagtaatacgactcactatagggagaaggctAAATAAACTCCTTTCCCTTCCTTTC-5′). Mass spectra were obtained via MassARRAY Compact MALDI-TOF (Sequenom) and their methylation ratios were generated using the Epityper software version 1.0 (Sequenom).

### Statistical analysis

Results are presented as mean ± SD of at least three independent experiments unless otherwise stated. Statistical analysis of group differences was performed using Student's two-tailed *t*-test. Statistical significance was defined as *P*<0.05.

## Results

### Characterization of the cultured CFs

First-passage neonatal rat CF cultures exhibited morphological characteristics that are typical for fibroblasts in culture and are positive for vimentin, a marker of fibroblast-like cells. These cell cultures did not contain desmin or factor VIII, markers of vascular smooth muscle and endothelial cells, or other structures that are considered typical for these cell cultures. These features indicated that these cells are CFs (Figure S1).

### TGF-β_1_ upregulated the collagen type I expression in CFs

For time dependence, CFs were divided into five groups according to different treatment time periods: CFs were treated with 10 ng/mL TGF-β_1_ for 0, 12, 24, 48, and 72 h. Figure S2A shows that the TGF-β_1_ treatment for 12 h to 72 h resulted in the upregulation of COL1A1 mRNA expression (*P*<0.05). The maximum increase was approximately twofold when the cells were treated with TGF-β_1_ for 48 h (*P*<0.01). The increase in cell-associated collagen type I is in accordance with the increased COL1A1 mRNA expression. Only the TGF-β_1_ treatment for 48 h can increase the collagen type I secretion (*P*<0.01). For dose dependence, CFs were divided into four groups according to different dose concentrations: CFs were treated with 0, 1, 10, and 50 ng/mL of TGF-β_1_ for 48 h. Figure S2B shows that the TGF-β_1_ treatment at 1 and 10 ng/mL resulted in the upregulation of COL1A1 mRNA expression and cell-associated collagen type I (*P*<0.05). TGF-β_1_ treatment at 50 ng/mL induced the expression of cell-associated collagen type I (*P*<0.01). The maximum increase was approximately twofold when the cells were treated with 10 ng/mL of TGF-β_1_ (*P*<0.01). However, only 10 ng/mL of TGF-β_1_ treatment increased collagen type I secretion (*P*<0.01).

### TGF-β_1_ and DNMT inhibitor upregulated the collagen type I expression in CFs

**Figure 1:**
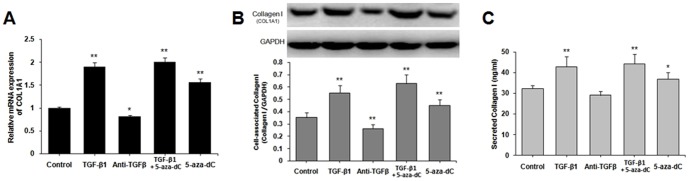
TGF-β_1_ and DNMT inhibitor induced the expression of collagen type I (COL1A1). Untreated cardiac fibroblasts (CFs) were cultured until near confluence was reached, and then starved for 12 h in serum-free DMEM. CFs were stimulated with 10 ng/mL of TGF-β_1_, 30 µg/mL of TGF-β-neutralizing antibody, 10 ng/mL TGF-β_1_+5 µM 5-aza-dC and 5 µM 5-aza-dC for 48 h. (A) Collagen type I (COL1A1) mRNA was determined via quantitative real-time PCR. (B) Cell-associated collagen type I was determined via Western blot. (C) Secreted collagen type I was determined via ELISA. Data are presented as mean ± SD (*n* = 3). *P<0.05, **P<0.01 (relative to the respective control).

5-Aza-dC, a DNMT inhibitor, was used to test whether or not a similar epigenetic regulation is involved in COL1A1 expression. CFs were treated with TGF-β_1_, TGF-β-neutralizing antibody, TGF-β_1_+5-aza-dC and 5-aza-dC. Figure 1 shows that TGF-β_1_ and 5-aza-dC significantly upregulated the mRNA expression of COL1A1 (*P*<0.01; Figure 1A), stimulated the cell-associated collagen type I synthesis (Figure 1B), and secreted collagen type I (Figure 1C) after 48 h of incubation. The maximum increase was approximately twofold in both mRNA and protein levels when the cells were treated with TGF-β_1_ and 5-aza-dC simultaneously. However, the TGF-β-neutralizing antibody downregulated COL1A1 and cell-associated collagen type I expressions (Figures 1A and B). No difference in the secreted collagen type I synthesis was observed between the control group and the TGF-β-antibody group (*P*>0.05; Figure 1C).

### TGF-β_1_ inhibited DNMT expression and activity

**Figure 2:**
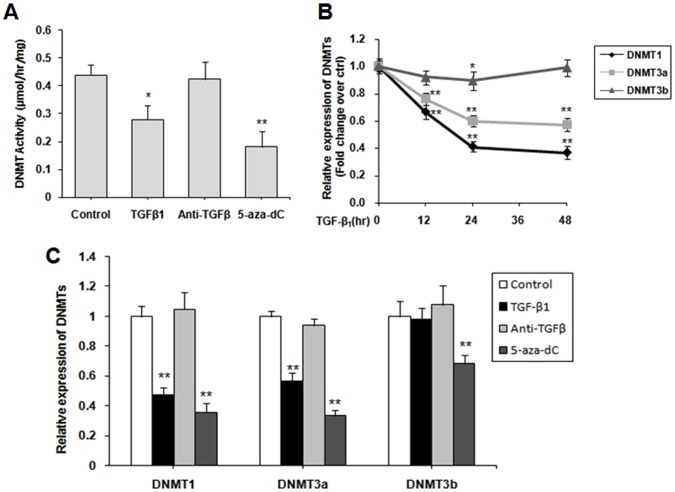
TGF-β_1_ inhibited the expression of DNMTs in cardiac fibroblasts (CFs). (A) CFs were starved for 12 h in serum-free DMEM, and then stimulated with 10 ng/mL TGF-β_1_, 30 µg/mL TGF-β-neutralizing antibody and 5 µM 5-aza-dC for 48 h. A DNMT activity assay kit was used to analyze the global DNMT activity. (B) CFs were treated with 10 ng/mL TGF-β_1_ for 0, 12, 24 and 48 h. The expression of three DNMTs were analyzed by quantitative real-time PCR. (C) CFs were treat as (A), qPCR was performed to quantify the relative mRNA levels of DNMT1, DNMT3a, and DNMT3b. Data were obtained from three independent experiments and expressed as mean ± SD (*n* = 3). *P <0.05, **P<0.01 (relative to the respective control).

We examined DNMT1, DNMT3a, and DNMT3b expressions in CFs to determine whether or not DNMTs regulate the COL1A1 expression through promoter methylation. The cells were stimulated with 10 ng/mL TGF-β_1_, 30 µg/mL TGF-β-neutralizing antibody and 5 µM 5-aza-dC for 48 h. Firstly, fresh nuclear extracts were prepared and assayed to determine global DNMT activity levels. The results showed that DNMT activity was significantly reduced in both TGF-β_1_-treated and 5-aza-dC-treated cells (*P*<0.05, *P* <0.01; Figure 2A). No difference was observed in TGF-β-neutralizing antibody group (*P* >0.05). Secondly, the respective DNMT isoform mRNA level was determined using quantitative real-time PCR. Figure 2C shows that the TGF-β_1_ treatment downregulated DNMT1 and DNMT3a expressions (*P* <0.01). No difference was observed for DNMT3b expression when treated with TGF-β_1_ (*P* >0.05). However, 5-aza-dC treatment downregulated all of the DNMTs expressions (*P* <0.01) and no difference was observed in TGF-β-neutralizing antibody group (Figure 2C). For time dependence, CFs were treated with 10 ng/mL TGF-β_1_ for 0, 12, 24 and 48 h. The mRNA of three DNMTs were analyzed. When treated with TGF-β_1_, DNMT1 and DNMT3a expressions downregulated from 0 to 48 h in a time-dependent manner (*P* <0.01; Figure 2B). The maximum decrease was observed in 48 h (*P* <0.01). However, no difference was observed for DNMT3b expression excecpt treated for 24 h (*P* <0.05).

### TGF-β_1_ induced the DNA demethylation of the COL1A1 promoter

**Figure 3:**
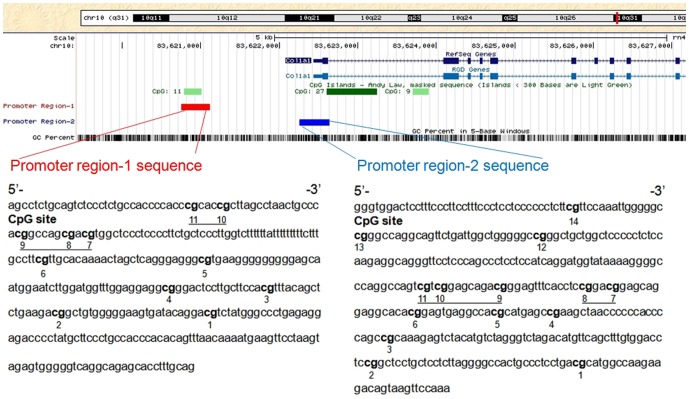
Schematic diagram of rat COL1A1 promoter. Two DNA fragments from rat distal and proximal promoters were amplified to analyze the methylation of the COL1A1 promoter. The fragments were labeled as promoter region-1 and promoter region-2. The location of promoter region-1 (–1682 bp to –1322 bp), promoter region-2 (–184 bp to +199 bp), and three CpG islands are indicated by a red bar, a blue bar, and three green bars, respectively. The start of exon 1 was considered as +1 of the sequence. PCR primers were designed based on the reverse complementary strands of these fragments. Promoter region-1 sequence represents 360 bp fragments and the CpG sites were numbered from 1 to 11 from the 3′-end to the 5′-end. Promoter region-2 sequence represents 384 bp fragments and the CpG sites were numbered from 1 to 14 from the 3′-end to the 5′-end. The numbers refer to the locations of the CpG sites. The underlined highlights correspond to the multiple CpG sites that were tested simultaneously.

**Figure 4:**
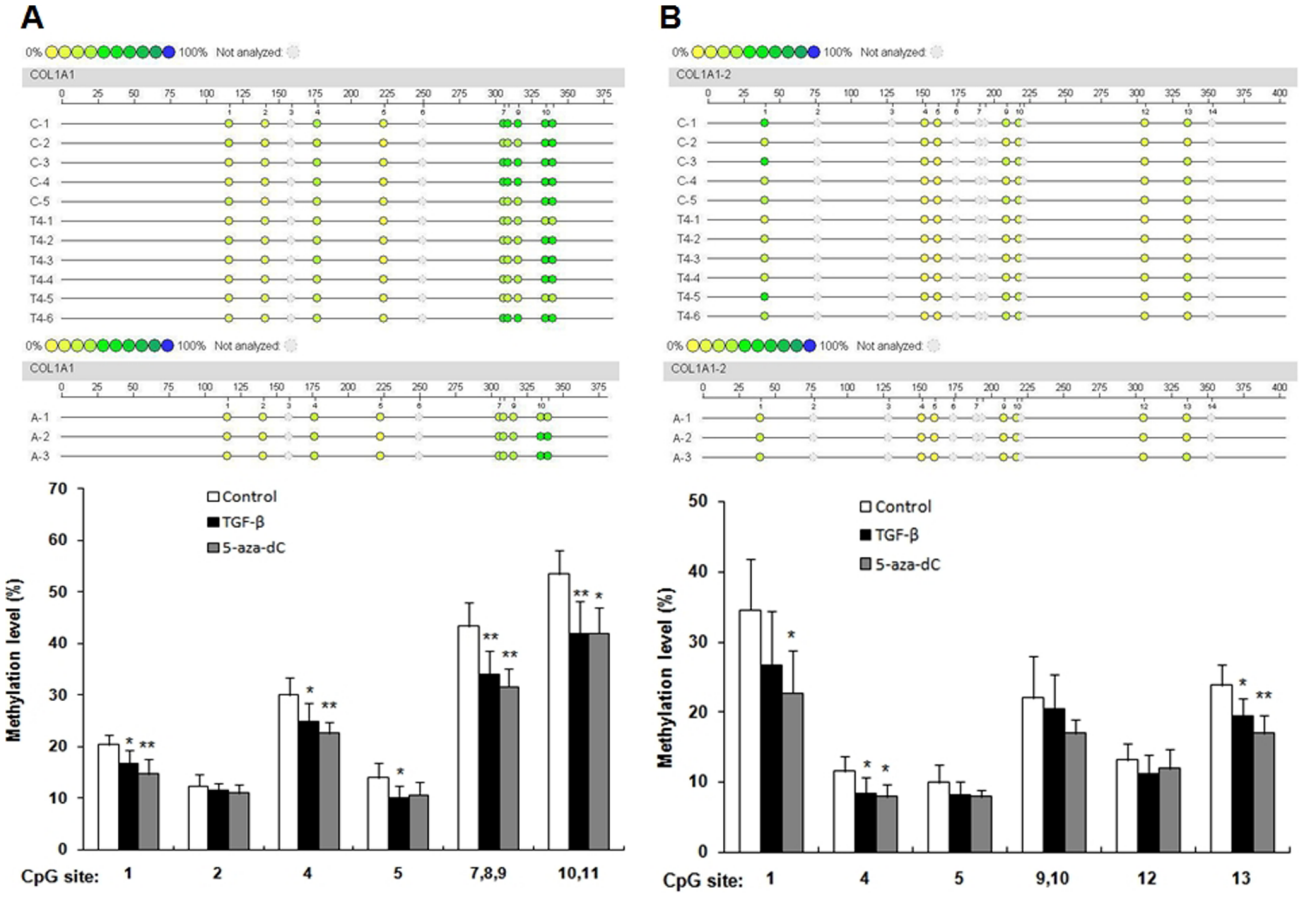
Methylation levels of the CpG sites in the COL1A1 promoter. The methylation levels of CpG sites in COL1A1 promoter regions from the control group (without treatment), TGF-β group (treated with 10 ng/mL TGF-β_1_ for 48 h) and 5-aza-dC group (treated with 5 µM 5-aza-dC for 48 h) were compared. Sequenom MassARRAY platform was used for the quantitative methylation analysis. The colors of each circle represent the methylation level of each corresponding CpG unit. Quantitative methylation analysis results are shown in a color scale: yellow (∼0% methylation), green (∼50% methylation), and dark blue (∼100% methylation). The white circles represent the missing data at a given CpG site. Mean methylation levels of CpG sites in (A) COL1A1 promoter region-1 and (B) COL1A1 promoter region-2. Data are expressed as mean ± SD∶ *n*_control_ = 5 (Sample: C-1 to C-5); *n*_TGF-β_  = 6 (Sample: T4-1 to T4-6); *n*_5-aza-dC_ = 3(Sample: A-1 to A-3). *P <0.05, **P <0.01 (relative to the respective control).

TGF-β_1_ can suppress DNMT expression and activity as well as increase COL1A1 expression. Thus, we investigated whether or not TGF-β_1_ induces COL1A1 expression through promoter demethylation. CFs were treated with 10 ng/mL TGF-β_1_ and 5 µM 5-aza-dC respectively for 48 h. Genomic DNA was extracted and subjected to bisulfite sequencing analysis. Figure 3 shows that the two DNA fragments from COL1A1 distal and proximal promoters were analyzed and labeled as promoter region-1 and promoter region-2, respectively. The methylation level of each CpG site within these two regions was evaluated. A total of 11 CpG sites in promoter region-1 were divided into 8 CpG site units; 14 CpG sites in promoter region-2 were divided into 11 CpG site units (Figure 3). The methylation levels varied at different CpG sites. For promoter region-1, the lowest methylation level (12%) was found at the 2^nd^ CpG site. The highest methylation level (53%) was found at the 10^th^ and 11^th^ CpG sites. Figure 4A shows that TGF-β_1_ can significantly reduce the methylation percentage across multiple CpG sites after 48 h of incubation. Demethylation significantly changed (22% of the level from the control samples) at 10^th^ and 11^th^ CpG sites. By contrast, no significant change was observed at the 2^nd^ CpG site (Figure 4A). When treated with 5-aza-dC, methylation percentage across multiple CpG sites in promoter region-1 were significantly reduced except the 2^nd^ and 5^th^ CpG sites. For promoter region-2, the lowest level (10%) and the highest level (35%) were found at the 5^th^ and the 1^st^CpG sites, respectively. Demethylation was observed only at the 4^th^ and 13^th^ CpG sites, whereas no significant change was observed at other CpG sites when treated with TGF-β_1_ (Figure 4B). 5-aza-dC treatment led to demethylation at the 1^st^, 4^th^ and 13^th^ CpG sites while no significant change was observed at other CpG sites.

## Discussion

In this study, we evaluated the epigenetic regulation of collagen type I in CFs. Our findings indicated that TGF-β_1_ upregulated the expression of collagen type I in mRNA and protein levels through the DNA demethylation of COL1A1 promoter regions and inhibition of DNMTs. To our knowledge, this study is the first to demonstrate the DNA methylation regulation of collagen type I in rat CFs.

The CF activation and the excessive deposition of collagen type I as well as other ECM proteins are a critical event in the progression of heart failure. The regulation of collagen type I expression has been extensively studied to understand the mechanism of fibrosis. Collagen type I is composed of three polypeptide chains transcribed from two separate genes (COL1A1 and COL1A2) with different promoters that require coordinate regulation [26]. The complete transcription of both genes is required in collagen type I synthesis. In this paper, we focused on COL1A1 gene. The TGF-β expression is increased in response to injury. Studies have described the basics of TGF-β signaling and its relationship to tissue repair as well as fibrosis [5], [27], [28]. In mammals, three TGF-β isoforms (TGF-β_1_, TGF-β_2_ and TGF-β_3_) have been identified and they exhibit similar but not identical biological properties. Evidence has indicated that TGF-β_1_ is an important regulator of ECM metabolism in different organ systems [29]. TGF-β_1_ is a key mediator of CF activation and has a major influence on collagen type I expression [7]. Our findings indicated that TGF-β_1_ can induce cell-associated and secreted collagen type I synthesis as well as upregulate COL1A1 mRNA expression.

Despite the discovery of an increasing number of *trans*-acting factors and *cis*-acting elements that control the COL1A1 gene expression [12], [13], the epigenetic regulation of its expression has not been widely studied. In the present study, we provided evidence that the COL1A1 expression in neonatal rat CFs treated with TGF-β_1_ is partly subject to epigenetic control. The CFs were also treated with 5-aza-dC, a strong inducer of DNA demethylation and an analog of cytosine, to investigate whether or not the COL1A1 mRNA expression is inactivated by methylation. 5-aza-dC irreversibly binds the methyltransferase enzymes as they attempt to methylate the cytosine analog when 5-aza-dC is incorporated into the DNA. This depletion of methyltransferases in the cell results in passive demethylation, which reactivates the epigenetically silenced genes [30]. Our experiments demonstrated that 5-aza-dC significantly induced the COL1A1 expression in CFs at both mRNA and protein levels, which is consistent with the results of TGF-β_1_ treatment. Based on these findings, TGF-β_1_ may have a similar mechanism with 5-aza-dC in COL1A1 regulation.

One mechanism of epigenetic regulation of gene expression is mediated by DNA methylation of CpG sites within promoters. This process can generally lead to gene silencing, a characteristic found in several human cancers, in which the expression of tumor suppressor genes is inhibited [31], [32]. The expression of COL1A1 is controlled by many factors, including a change in the DNA methylation status [20]-[24], [33]. Our experiments also demonstrated that TGF-β_1_ has an effect similar to 5-aza-dC in COL1A1 regulation, indicating that an additional mechanism by which TGF-β_1_ can regulate the COL1A1 gene expression by DNA methylation suppression occurs. Evidence has indicated that cytosine methylation at the CpG sequence suppresses gene expression, whereas demethylation activates gene expression. This relationship is clear, particularly when the change in DNA methylation occurs in the promoter region, called the “CpG island,” where CpG is present at a high frequency [34], [35]. A major TGF-β_1_ response element has been reported at position –1624 in the rat distal promoter of the COL1A1 gene [36], but the functional importance of this site has subsequently been questioned [37]. Recent studies demonstrated that TGF-β-responsive sequences of the COL1A1 promoter are located in the proximal promoter between –174 and –84 bp from the initiation site of transcription, which contains a binding site for Sp1 [11]. In the present study, two DNA fragments from the rat COL1A1 promoter region (promoter region-1 and promoter region-2) were analyzed. Promoter region-1 (–1682 bp to –1322 bp) is from the distal promoter and located in a CpG island, whereas promoter region-2 (–184 bp to +199 bp) is from the proximal promoter. Our data indicated that TGF-β_1_ can significantly reduce the methylation percentage across multiple CpG sites in the rat COL1A1 promoter, particularly in promoter region-1 (5 out of 6 CpG site units). It is likely that demethylation at many CpG sites, rather than a specific CpG, in the promoter region is involved in the alteration of COL1A1 gene expression, probably through alteration in chromatin structure [34]. Promoter region-1 is located in a CpG island, indicating that this region may be more sensitive to DNA methylation than promoter region-2. DNMT expression was also evaluated to examine if TGF-β_1_ can regulate COL1A1 expression through DNA methylation and confirm whether or not the effects of manipulating DNA methylation can be altered by TGF-β_1_.

Mammalian cells have three DNMTs, namely, DNMT1, DNMT3a, and DNMT3b that are responsible for DNA methylation. DNMT1 is a maintenance-type methyltransferase, which is responsible for copying DNA methylation patterns during DNA replication; DNMT3a and DNMT3b are important in de novo methylation [38], [39]. TGF-β_1_ can downregulate all of the three DNMTs and induce Foxp3 expression in T-cells [40]. In liver cancer cells, TGF-β_1_ can regulate CD133 expression through the inhibition of DNMT1 and DNMT3b expressions; TGF-β_1_ stimulation results in a significant demethylation of CD133 promoter-1 [41]. Similarly, TGF-β_1_ treatment inhibits DNMT1 and DNMT3a expressions and subsequently induces the α-smooth muscle actin expression in rat lung fibroblasts [42]. In the present study, we found that TGF-β_1_ significantly inhibited the global DNMT activity and downregulated the mRNA expression of DNMT1 and DNMT3a in a time-dependent manner. Our results are in agreement with those in previous reports, which showed that TGF-β_1_ can inhibit DNMT expression. However, this inhibitory effect of TGF-β_1_ is inconsistent with that found in aggressive prostate cancer, in which TGF-β_1_ induces DNMT expression [43]. The basis for this difference is unclear but may be related to the different cell types and/or experimental conditions used.

In summary, our findings indicated that TGF-β_1_ can induce the synthesis of cell-associated and secreted collagen type I. TGF-β_1_ can also upregulate the COL1A1 mRNA expression. Furthermore, TGF-β_1_-induced COL1A1 expression occurred through the inhibition of global DNMT activity as well as downregulation of DNMT1 and DNMT3a expressions, thereby leading to the demethylation of the rat COL1A1 promoter. These findings described the mechanism by which TGF-β_1_ regulates the collagen type I expression through COL1A1 promoter demethylation. However, this study only examined the neonatal rat CFs. Thus, the epigenetic characteristics may differ from those in adult rats. Further studies on methylation regulation via the first intronic region in COL1A1 expression should be considered in the future.

## Supporting Information

Figure S1 **Characterization of cardiac fibroblasts.** First-passage cardiac fibroblasts from neonatal Sprague-Dawley rats were cultured until near confluence was reached. The cells were washed, fixed, and immunocytochemically stained with antibodies against vimentin, desmin, and factor VIII. Scale bar  =  20 µm.
(TIF)

Figure S2 **Transforming growth factor-beta 1 (TGF-β_1_) upregulated the expression of collagen type I (COL1A1).** Untreated cardiac fibroblasts (CFs) were cultured until near confluence was reached, and then starved for 12 h in serum-free DMEM. Collagen type I (COL1A1) mRNA was determined via quantitative real-time PCR. Cell-associated collagen type I was determined via Western blot and secreted collagen type I was determined via ELISA. (A) CFs were stimulated with 10 ng/mL of TGF-β_1_ from 0 h to 72 h. (B) CFs were stimulated with 0 ng/mL to 50 ng/mL of TGF-β_1_ for 48 h. Data are presented as mean ± SD (*n*  =  3). *P<0.05, **P<0.01 (relative to the respective control).
(TIF)
