# Supplementary figures and images for: Transforming Growth Factor β1 Induces the Expression of Collagen Type I by DNA Methylation in Cardiac Fibroblasts
Source: PLoS One. 2013 Apr 1;8(4):e60335. doi: 10.1371/journal.pone.0060335 (PMC3613378; doi:10.1371/journal.pone.0060335)

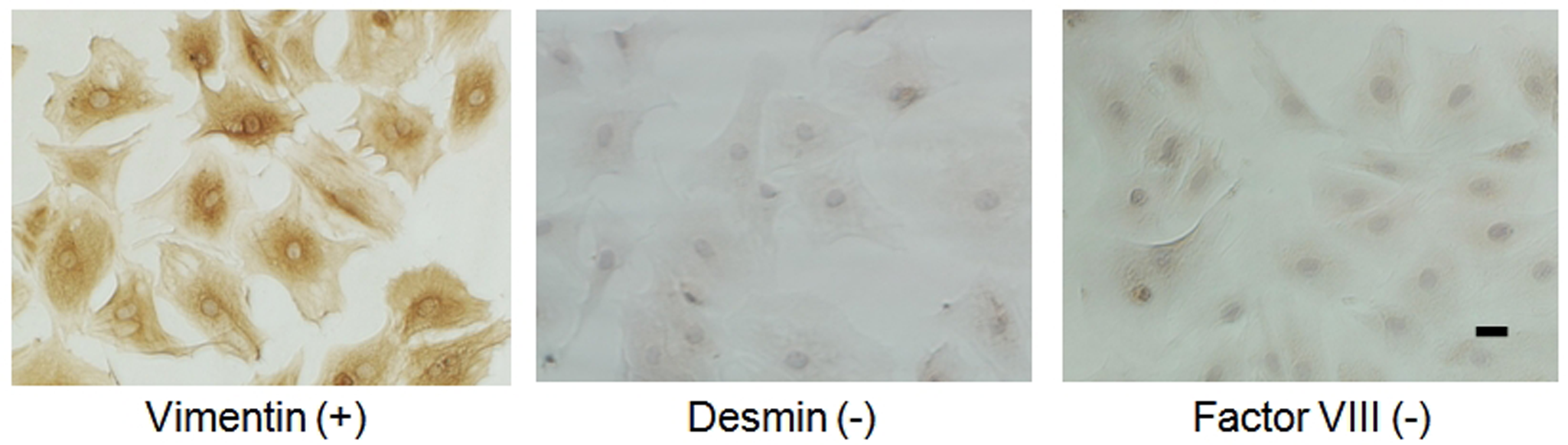

Supplement: Figure S1 — Characterization of cardiac fibroblasts. First-passage cardiac fibroblasts from neonatal Sprague-Dawley rats were cultured until near confluence was reached. The cells were washed, fixed, and immunocytochemically stained with antibodies against vimentin, desmin, and factor VIII. Scale bar = 20 µm. (TIF) [file pone.0060335.s001.tif]

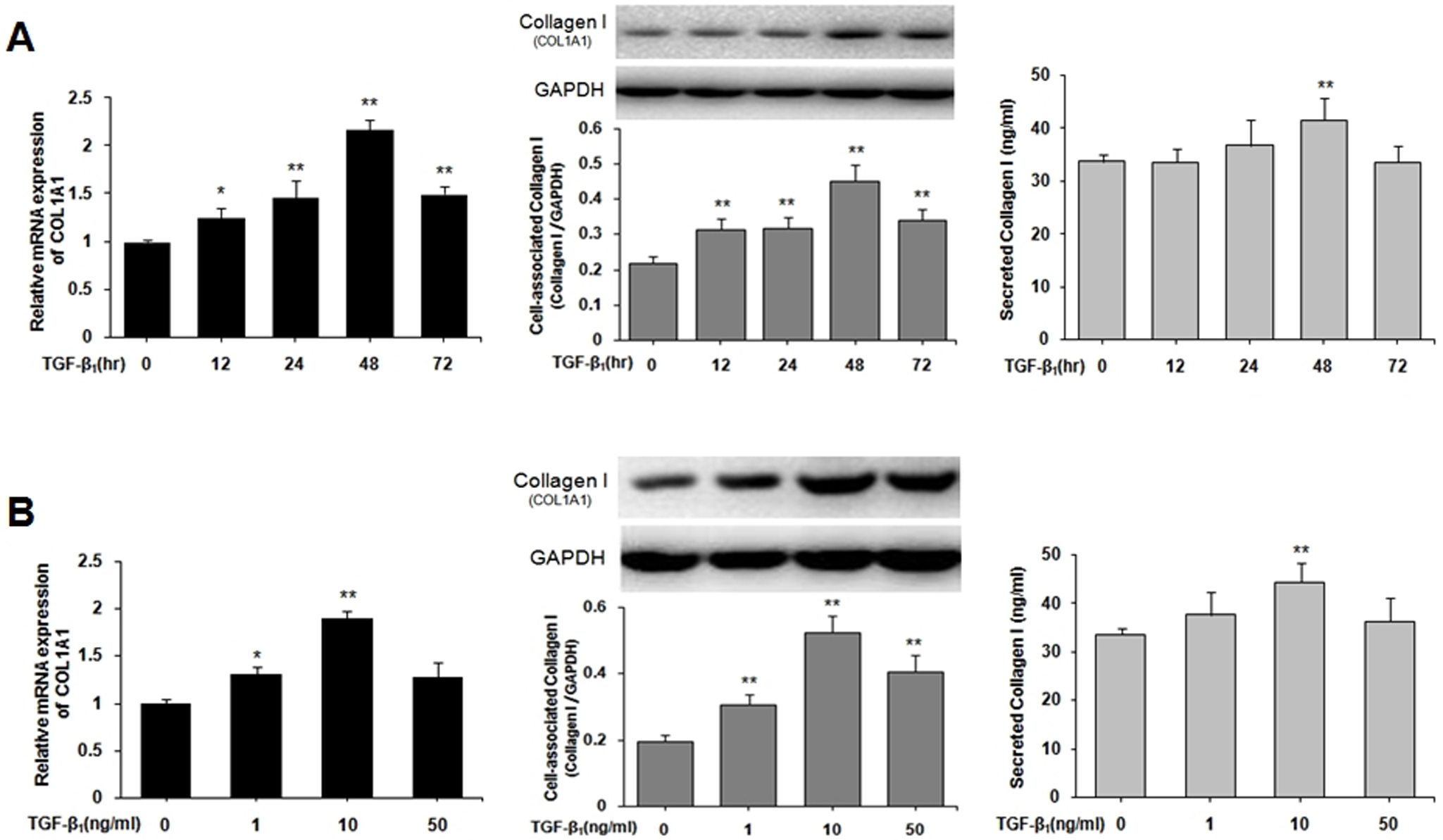

Supplement: Figure S2 — Transforming growth factor-beta 1 (TGF-β1) upregulated the expression of collagen type I (COL1A1). Untreated cardiac fibroblasts (CFs) were cultured until near confluence was reached, and then starved for 12 h in serum-free DMEM. Collagen type I (COL1A1) mRNA was determined via quantitative real-time PCR. Cell-associated collagen type I was determined via Western blot and secreted collagen type I was determined via ELISA. (A) CFs were stimulated with 10 ng/mL of TGF-β1 from 0 h to 72 h. (B) CFs were stimulated with 0 ng/mL to 50 ng/mL of TGF-β1 for 48 h. Data are presented as mean ± SD (n = 3). *P<0.05, **P<0.01 (relative to the respective control). (TIF) [file pone.0060335.s002.tif]
